# A systematic review and meta-analysis of integrated studies on antimicrobial resistance in Vietnam, with a focus on *Enterobacteriaceae*, from a One Health perspective

**DOI:** 10.1016/j.onehlt.2022.100465

**Published:** 2022-11-19

**Authors:** Doan Hoang Phu, Tuempong Wongtawan, Dinh Bao Truong, Nguyen Van Cuong, Juan Carrique-Mas, Thotsapol Thomrongsuwannakij

**Affiliations:** a Akkhraratchakumari Veterinary College, Walailak University, Nakhon Si Thammarat 80160, Thailand; b College of Graduate Studies, Walailak University, Nakhon Si Thammarat 80160, Thailand; c Faculty of Animal Science and Veterinary Medicine, Nong Lam University, Ho Chi Minh City 70000, Viet Nam; d Centre for One Health, Walailak University, Nakhon Si Thammarat 80160, Thailand; e Centre of Excellence Research for Melioidosis and other Microorganism, Walailak University, Nakhon Si Thammarat 80160, Thailand; f Oxford University Clinical Research Unit, Ho Chi Minh City 70000, Viet Nam; g Ausvet PTY LTD, Bruce ACT 2617, Canberra, Australia; h Food and Agriculture Organization of the United Nations, Ha Noi 10000, Viet Nam

**Keywords:** Antimicrobial resistance, *Enterobacteriaceae*, One Health, Systematic review, Meta-analysis, Vietnam

## Abstract

Vietnam is a low- and middle-income country (LMIC), a primary food producer, and an antimicrobial resistance (AMR) hotspot. AMR is recognized as a One Health challenge since it may transfer between humans, animals and the environment. This study aimed to apply systematic review and meta-analysis to investigate the phenotypic profiles and correlations of antimicrobial-resistant *Enterobacteriaceae* across three compartments: humans, animals and the environment in Vietnam. A total of 89 articles found in PubMed, Science Direct, and Google Scholar databases were retrieved for qualitative synthesis. *E. coli* and non-typhoidal *Salmonella* (NTS) were the most common bacterial species in studies of all compartments (60/89 studies). Among antimicrobials classified as critically important, the resistance levels were observed to be highest to quinolones, 3rd generation of cephalosporins, penicillins, and aminoglycosides. Of 89 studies, 55 articles reported the resistance prevalence of *E. coli* and NTS in healthy humans, animals and the environment against ciprofloxacin, ceftazidime, ampicillin, gentamicin, sulfamethoxazole-trimethoprim, chloramphenicol was used for meta-analysis. The pooled prevalence was found highest in *E. coli* against ampicillin 84.0% (95% CI 73.0–91.0%) and sulfamethoxazole-trimethoprim 66.0% (95% CI 56.0–75.0%) while in NTS they were 34.0% (95% CI 24.0–46.0%), 33.0% (95% CI 25.0–42.0%), respectively. There were no significant differences in the pooled prevalence of *E. coli* and NTS to these antimicrobials across healthy humans, animals and the environment, except for ceftazidime-resistant *E. coli* (χ^2^ = 8.29, *p* = 0.02), chloramphenicol-resistant *E.coli* (χ^2^ = 9.65, *p* < 0.01) and chloramphenicol-resistant NTS (χ^2^ = 7.51, p = 0.02). Findings from the multiple meta-regression models indicated that the AMR levels in *E. coli* (β = 1.887, *p* < 0.001) and the North (β = 0.798, *p* = 0.047) had a higher fraction of AMR than NTS and other regions of Vietnam. The outcomes of this study play an important role as the baseline information for further investigation and follow-up intervention strategies to tackle AMR in Vietnam, and more generally, can be adapted to other LMICs.

## Highlights

• This review reports the high AMR levels of *E. coli* and NTS in Vietnam, especially to critically important antimicrobials.
• There was a marginal difference in the AMR levels in the three compartments of humans, animals and the environment.
• The North of Vietnam and *E. coli* had higher levels of AMR compared with other regions of Vietnam and NTS.
• The results highlight the gaps in research for further investigation and future strategies to mitigate AMR in Vietnam.
• Implementation of AMR surveillance- under the One Health approach is required to tackle the AMR problems in Vietnam.

## 1 Introduction

Antimicrobial resistance (AMR) has been declared by the World Health Organization (WHO) as a top ten global public health threat [1]. AMR leads to ineffective treatment of infectious diseases, resulting in increased mortality rates, economic losses and compromised food security [2]. Annually, it is estimated that around 5 million persons die due to AMR bacterial infections, with LMICs bearing the heaviest burden [3].

Bacterial species belonging to the *Enterobacteriaceae* family are included in AMR surveillance programmes worldwide. In food animal production systems, both commensal *E. coli* and non-typhoidal *Salmonella* (NTS) are commonly included in monitoring programs [4,5]. In addition to NTS and *E. coli* (both commensal and pathogenic), other *Enterobacteriaceae* including *Klebsiella* spp., and *Shigella* spp., have been monitored for their AMR profiles in human populations [6]. Resistance to the last-resort antimicrobial drugs (such as carbapenem and colistin) among *Enterobacteriaceae* has increased globally, especially in LMICs [[7], [8], [9]]. Increased resistance among *Enterobacteriaceae* is partly associated with the overuse of antimicrobials in animal production systems [[10], [11], [12], [13]].

Vietnam is currently classified by the World Bank as a low- and middle-income country. The agricultural sector (including animal production) represents 15.0% of the national gross domestic product [14]. Vietnam is also a hotspot of AMR [15,16]. A significant driver of AMR in Vietnam and elsewhere is the indiscriminate use of antimicrobials in animal production, which is estimated to account for ∼70.0% of the total consumption of antimicrobials in the country [17]. In the last decade, annual poultry production in the country has increased from 2010 to 2020 (from 300.5 to 512.7 million chickens, a 70.6% increase). Pig populations also increased after the incursion of African Swine Fever in January 2019; the total population resumed by 13.3% in 2020, reaching 22.0 million pigs from 19.6 million pigs in 2019 [18]. The increased antimicrobial consumption is linked to the intensification of animal production, especially for prevention and growth promotion purposes [19]. The most commonly identified antimicrobial-resistant bacterial species belong to the *Enterobacteriaceae* family*,* mainly *E. coli*, followed by non-typhoidal *Salmonella* and *Klebsiella* spp. [20].

Since AMR bacteria and AMR-encoding genes can flow between humans, animals and the environment, applying a One Health approach is crucial to tackling this problem [21]. The present study aims to describe and analyze the phenotypic profiles and correlations of antimicrobial-resistant *Enterobacteriaceae* in Vietnam's three One Health compartments (humans, animals and the environment). Findings from this review highlight the gaps in research for further investigation and future strategies to mitigate AMR in Vietnam. These findings may potentially be used as a model for other LMICs in the region.

## 2 Materials and methods

### 2.1 Study protocol

A systematic review was performed according to the PRISMA guideline [22], and all 27 checklist items were addressed in the study (Supplementary File 1).

### 2.2 Search strategy

Research articles were identified through (1) PubMed, (2) ScienceDirect, (3) Google Scholar, and (4) Vietnamese journal websites. Boolean Logic tools with the connectors ‘AND’, ‘OR’ were applied to search the articles. The keywords for advanced search used in PubMed were: (antimicrobial resistance OR antibiotic resistance) AND (human OR animal OR environment) AND (*Enterobacteriaceae* OR *E. coli* OR *Escherichia coli* OR *Salmonella* OR *Klebsiella* OR *Proteus* OR *Shigella* OR *Enterobacter* OR *Citrobacter* OR *Cronobacter*) AND (livestock OR poultry OR cattle OR swine OR pig OR chicken OR fish OR shrimp OR shellfish) AND (meat OR pork OR beef OR milk) AND (environment OR soil OR water OR wastewater OR sludge OR vegetable OR drainage) AND (Vietnam OR Vietnamese); and in ScienceDirect: (antimicrobial OR antibiotic) AND (resistance OR susceptibility OR sensitivity) AND (Vietnam OR Vietnamese). Furthermore, hand-searching in Google Scholar, Vietnamese journal websites, and reference lists of selected articles were also applied to have a higher range of articles that satisfy eligibility criteria.

### 2.3 Selection of publications

The selection of articles for review was completed based on three stages: title, abstract and full text. Articles included must describe the prevalence of AMR in *Enterobacteriaceae*, and sample collection was from (1) humans (healthy/ diseased humans in hospitals); (2) animals (both terrestrial and aquatic) healthy and diseased; animal food products (meat, egg, and milk); (3) the environment (water, drainage, soil, sludge, vegetables, commercial feed, and wildlife animals). Additionally, all studies must apply antimicrobial susceptibility testing methods according to the Clinical and Laboratory Standards Institute (CLSI) guidelines. Articles that did not contain sufficient information on bacterial species, phenotypic resistance prevalence data or disaggregated data by bacterial species were excluded. Moreover, review articles, book chapters, conference abstracts, letters, and articles written in languages other than English and duplicated among databases were also excluded.

In order to minimize selection bias, the study quality assessment of included studies was appraised using the Joanna Briggs Institute (JBI) Critical Appraisal Checklist for studies reporting prevalence data [23]. A total number of 9 questions in the checklist were accessed by two independent reviewers based on the JBI guideline with the following answers: ‘yes’, ‘no’, ‘unclear’, and ‘not applicable’. Only studies appraised with ‘yes’ for all questions were included. If there were discrepant results between reviewers for any question, a third independent reviewer participated in the assessment process; studies were included when there was a consensus of ‘yes’ from two out of three reviewers.

### 2.4 Data extraction

Data from each retrieved publication were reviewed and the following information were extracted and saved in a Microsoft Excel 2019 spreadsheet: (1) region of Vietnam (North/ Center/ South); (2) publication year; (3) host (humans/ animals/ environment/ integrated studies between animals, humans and environment); (4) type of sample (feces, stool, rectal swab/ wound/ nasal swabs/ blood/ pus/ urine/ vaginal/ meat/ milk/ eggs/ water/ soil/ vegetables, etc.); (5) bacterial species (*E.coli*, *Samonella* spp., *Klebsiella* spp*., Proteus* spp*., Shigella* spp*., Enterobacter* spp., *Citrobacter* spp., *Cronobacter* spp*.,* etc.); (6) number of isolates; (7) animal species (chicken or chicken meat/ pig or pork/ cattle or beef/ fish/ shrimp/ rodent/ shellfish, etc.); (8) laboratory methods for antimicrobial susceptibility testing (minimum inhibitory concentration or disk diffusion); (9) prevalence (%) of resistant bacteria to antimicrobials, antimicrobial agents and antimicrobial classes were classified by WHO (highest priority critically important/ high priority critically important/ highly important/important) [24]; (10) prevalence of multi-drug resistance (MDR) isolates, which were resistant to at least one antimicrobial agent of more than three antimicrobial classes [25]. The data from included studies were extracted by two independent reviewers to reduce the information bias. The results were rechecked by a third reviewer. The data were ready for analysis after reaching a consensus among all reviewers.

### 2.5 Statistical analyses

Only studies that included data on AMR in bacterial species covering at least two articles of three compartments (humans, animals and environment) were chosen for meta-analysis. Heterogeneity assessment of selected studies for meta-analysis was applied using inverse variance index (*I*^*2*^)*,* an index >75.0% and a *p*-value <0.05 is considered as significant heterogeneity [26]. One recognized problem in meta-analysis with prevalence data derives from studies reporting very small or large prevalence; such estimates have minimal variance and, therefore, a greater weight (pooling effect size) [27]. Therefore, the logit-transformed proportions were applied using a generalised linear mixed-effect model (GLMM), which includes a random-effect model to identify the within-studies variance (V_Yi_) and estimate of between-studies variance (*τ*^*2*^*)* [28]. Moreover, subgroup analysis was applied in each selected antimicrobial for meta-analysis of AMR prevalence to identify the significant difference among study compartments of animals, humans, and the environment with AMR prevalence. Besides, multiple meta-regression models were used to identify the risk factors associated with the MDR prevalence with bacterial species, study compartments, study area, and year of publication as explanatory variables. Multiple meta-regression was built using a stepwise approach to select the final model. Variables of a multivariable model with *p*-value <0.05 were considered as a significant association with the MDR prevalence. The results of the meta-analysis were displayed by forest plots. Publication bias in the meta-analysis was applied using both methods, including contour-enhanced funnel plots and Egger's regression test [29]. All statistical analyses were applied by R software with the packages ‘meta’ and “metafor” used for meta-analysis, subgroup analyses, and multiple meta-regression; package ‘tidyverse’ used for testing publication bias; and “ggplot2” for visualization of the study results.

## 3 Results

### 3.1 Study selection

**Fig. 1:**
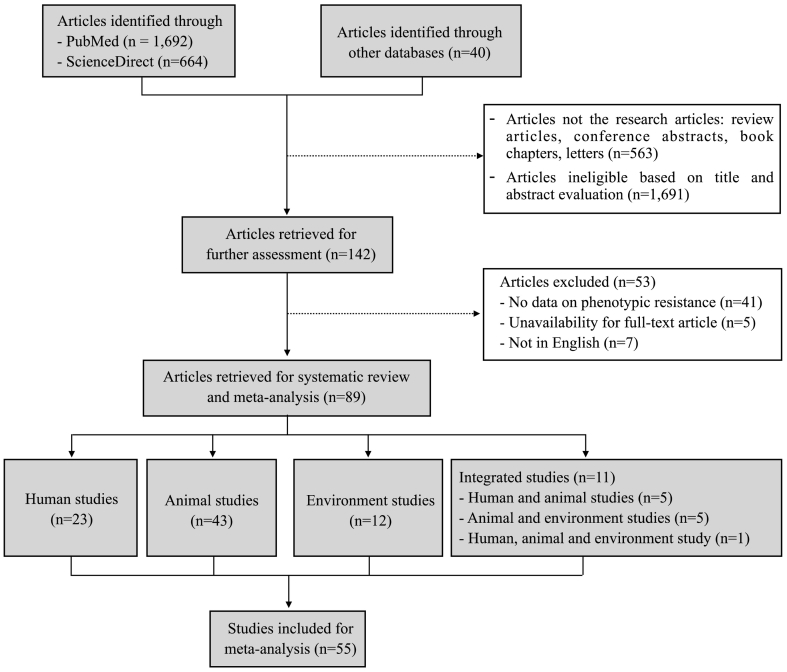
PRISMA flow diagram of the study selection.

A total of 2396 articles were initially identified. Of those, 142 were further selected after excluding non-research articles (*n* = 563) and ineligible articles (*n* = 1691) based on the evaluation of the title and abstract. Thereafter, 53 articles were further excluded because they contained no phenotypic AMR data (*n* = 41), the full-text was not available (n = 5), or they were published in languages other than English (*n* = 7). Finally, 89 articles were selected for the systematic review, including 23 human studies, 43 studies of animals and food, 12 environmental studies, and 11 studies on AMR in isolates from more than one compartment (animals, humans or the environment). Furthermore, out of 89 articles, we selected the articles reporting data on the AMR prevalence of each antimicrobial agent with at least two studies for each bacterial species in all three compartments of humans, animals and environments. Finally, there was a total of 55 articles used for meta-analysis. All 89 selected studies are presented in Supplementary File 2. The process of study selection followed the PRISMA flow diagram and is shown in Fig. 1.

### 3.2 Qualitative syntheses of selected studies

**Fig. 2:**
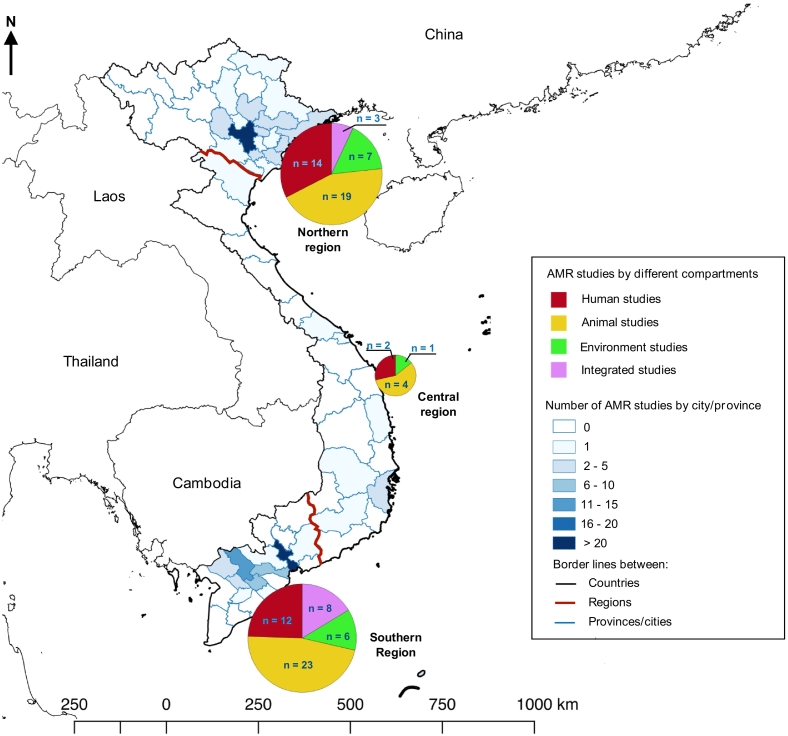
Study sites and the number of articles in each compartment in the review.

The description of selected studies for qualitative syntheses is presented in Table 1. All 89 selected studies were published between 2002 and 2022. Most selected studies were published in last recent years over the 2016–2022 period (54/89, 60.7%) and mostly conducted in the southern (43/89, 48.3%) and the northern regions of Vietnam (37/89, 41.6%). The study sites and the number of articles published in three different regions are illustrated in Fig. 2.

**Table 1:** Number of studies in each category.

Description of selected studies	Human studies (*n* = 23) (25.8)	Animal studies (*n* = 43) (48.3)	Environment studies (*n* = 12) (13.5)	Integrated studies (*n* = 11) (12.4)			Total (*n* = 89) (%)
				Human and animal (n = 5) (5.6)	Animal and environment (n = 5) (5.6)	Human, animal and environment (n = 1) (1.1)	
Bacterial species							
*E. coli*	7 (7.9)	26 (29.2)	6 (6.7)	2 (2.2)	1 (1.1)	_	42 (47.2)
*Salmonella* spp.	3 (3.4)	14 (15.7)	5 (5.6)	3 (3.4)	3 (3.4)	1 (1.1)	29 (32.6)
*Klebsiella* spp.	2 (2.2)	_	_	_	_	_	2 (2.2)
*Shigella* spp.	1 (1.1)	_	_	_	_	_	1 (1.1)
*E. coli, Salmonella* spp.	_	3 (3.4)	_	_	1 (1.1)	_	4 (4.5)
*E. coli, Klebsiella* spp.	3 (3.4)	_	_	_	_	_	3 (3.4)
*E. coli, Shigella* spp.	3 (3.4)	_	_	_	_	_	3 (3.4)
*E. coli, Salmonella* spp., *Shigella* spp.	1 (1.1)	_	_	_	_	_	1 (1.1)
*E. coli, Klebsiella* spp., *Enterobacter* spp.	1 (1.1)	_	_	_	_	_	1 (1.1)
*E. coli, Klebsiella* spp., *Proteus* spp.	1 (1.1)	_	_	_	_	_	1 (1.1)
*E. coli, Klebsiella* spp., *Enterobacter* spp., *Proteus* spp.	1 (1.1)	_	_	_	_	_	1 (1.1)
*E. coli, Salmonella* spp., *Klebsiella* spp., *Enterobacter* spp., *Cronobacter* spp.	_	_	1 (1.1)	_	_	_	1 (1.1)

Year of publication							
2002–2005	3 (3.4)	_	_	_	_	_	3 (3.4)
2006–2010	5 (5.6)	7 (7.9)	2 (2.2)	2 (2.2)	_	_	16 (18.0)
2011–2015	4 (4.5)	9 (11.2)	1 (1.1)	_	2 (2.2)	_	16 (18.0)
2016–2020	8 (9.0)	21 (23.6)	7 (7.9)	3 (3.4)	2 (2.2)	1 (1.1)	42 (47.2)
2021–2022	3 (3.4)	6 (6.7)	2 (2.2)	_	1 (1.1)	_	12 (13.5)

Study area							
Northern region	11 (12.4)	17 (19.1)	6 (4.5)	1 (1.1)	2 (2.2)	_	37 (41.6)
Central region	_	3 (3.4)	_	_	_	_	3 (3.4)
Southern region	9 (10.1)	21 (23.6)	5 (5.6)	4 (4.5)	3 (3.4)	1 (1.1)	43 (48.3)
Northern and Southern regions	1 (1.1)	1 (1.1)	_	_	_	_	2 (2.2)
Northern, Central and Southern regions	2 (2.2)	1 (1.1)	1 (1.1)	_	_	_	4 (4.5)

Sample type							
Stool/ faecal/ rectal swab	17 (19.1)	22 (24.7)	1 (1.1)	3 (3.4)	_		43 (48.3)
Blood	4 (4.5)	_	_	_	_		4 (4.5)
Carcass rinse/ meat (animals)	_	19 (21.3)	_	_	1 (1.1)		20 (22.5)
Sludge/wastewater	_	_	3 (3.4)	_	_		3 (3.4)
Vegetables/ fruit/ edible ice	_	_	4 (4.5)	_	_		4 (4.5)
Mixed sample collection	2 (2.2)	2 (2.2)	4 (4.5)	2 (2.2)	4 (4.5)	1 (1.1)	15 (16.9)

Antimicrobial susceptibility testing method							
Disc diffusion (DD)	9 (10.1)	29 (32.6)	7 (7.9)	4 (4.5)	4 (4.5)	1 (1.1)	54 (60.7)
Minimum Inhibitory Concentration (MIC)	11 (12.4)	12 (13.5)	4 (4.5)	1 (1.1)	_	_	28 (31.5)
Both DD and MIC	3 (3.4)	2 (2.2)	1 (1.1)	_	1 (1.1)	_	7 (7.9)

*E. coli* was the most common species in the selected studies (42/89, 47.2%), followed by *Salmonella* spp. (29/89, 32.6%), *Klebsiella* spp. (2/89, 2.2%) and *Shigella* spp. (1/89, 1.1%). There were 15 studies (16.9%) covering more than one bacterial species: *E. coli* and *Salmonella* spp. (4/89, 4.5%), *E. coli* and *Klebsiella* spp. (3/89, 3.4%), *E. coli* and *Shigella* spp. (3/89, 3.4%), and *E. coli, Salmonella* spp., *Klebsiella* spp., *Enterobacter* spp., and *Cronobacter* spp. (1/89, 1.1%). Most (54/89, 60.7%) selected studies applied the agar disc diffusion method for antimicrobial susceptibility testing, and other studies (28/89, 31.5%) used agar/broth methods to determine the minimum inhibitory concentration (MIC) of antimicrobial substances.

In the compartments of humans and animals, the new subsets were split into healthy/ diseased groups. Regarding 29 human studies (23 human studies only, 6 integrated studies related to humans), we found that *E. coli* were the common bacteria in humans, 5 and 10 studies in the healthy and diseased group, while NTS was found in 2 studies and 4 studies in healthy and diseased human studies. Most other bacterial species (*Klebsiella* spp., *Enterobacter* spp., *Shigella* spp., *Proteus* spp., and ESBL-producing *E. coli*) were conducted in diseased humans. In animal studies, there were 21 and 25 studies performed in *E.coli* and NTS, 4 studies worked on the ESBL-producing *E. coli* in healthy animals. In the group of diseased animal studies, only 3 studies conducted *E. coli* in diarrheal pigs. Similarly, in the environment compartment, *E. coli* (9 studies) and NTS (9 studies) were primarily found in environmental studies. The details of studies by bacterial species in each compartment of human, animal, and environment are shown in Table 2.

**Table 2:** Total of studies by bacterial species in each compartment of human, animal, and environment.

Bacteria	Human studies		Animal studies		Environment studies
	Healthy	Diseased (in hospitals)	Healthy animals/animal food products	Diseased	
*E. coli*	5 [[30], [31], [32], [33], [34]]	10 [[35], [36], [37], [38], [39], [40], [41], [42], [43], [44]]	21 [12,13,[45], [46], [47], [48], [49], [50], [51], [52], [53], [54], [55], [56], [57], [58], [59], [60], [61], [62], [63]]	3 [[64], [65], [66]]	9 [52,58,[67], [68], [69], [70], [71], [72], [73]]
ESBL-producing *E. coli*	_	2 [74,75]	4 [[76], [77], [78], [79]]	_	_
ESBL/pAmpC-producing *E. coli*	_	_	2 [80,81]	_	_
Cephalosporin-resistance *E. coli*	1 [82]	_	1 [82]	_	_
*mcr1*_positive *E. coli*	1 [83]	_	1 [84]	_	_
Non-typhoidal *Salmonella (NTS)*	2 [85,86]	4 [37,85,87,88]	25 [53,[57], [58],^60^,^85^,^86^,[88], [89], [90], [91], [92], [93], [94], [95], [96], [97], [98], [99], [100], [101], [102], [103], [104], [105], [106]]	_	9 [58,73,97,104,[107], [108], [109], [110], [111]]
Typhoidal *Salmonella*	_	3 [87,112,113]	_	_	_
*Enterobacter* spp.	_	2 [35,36]	_	_	1 [73]
*Cronobacter* spp.	_	_	_	_	1 [73]
*Klebsiella* spp.	1 [33]	5 [35,36,38,114,115]	_	_	1 [73]
ESBL-producing *Klebsiella*	_	2 [74,75]	_	_	_
*Proteus* spp.	_	1 [36]	_	_	_
ESBL-producing *Proteus*	_	1 [75]	_	_	_
*Shigella* spp.	_	5 [37,[40], [41], [42],^116^]	_	_	_

ESBL-producing *E. coli/ Klebsiella/ Proteus*: Extended-spectrum *β*-lactamase- (ESBL-) producing *Escherichia coli/ Klebsiella/ Proteus*; ESBL/pAmpC-producing *E. coli*: Extended-spectrum *β*-lactamase- (ESBL-) and plasmid-mediated AmpC *β*-lactamase- (pAmpC-) producing *Escherichia coli*; *mcr1*_positive *E. coli*: Colistin-resistant *Escherichia coli* harboring *mcr-1* gene.

### 3.3 Quantitative syntheses of selected studies

**Fig. 3:**
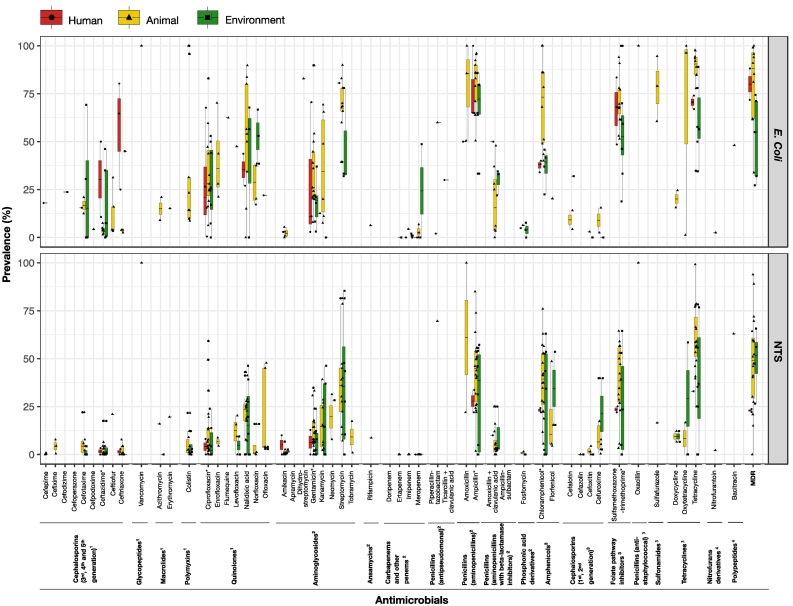
The AMR prevalence of *E. coli* and NTS in healthy human, healthy animal/animal food products and environment studies. ^1^highest priority critically important antimicrobials, ^2^high priority critically important antimicrobials, ^3^highly important antimicrobials, ^4^important antimicrobials, *antimicrobials of studies used for meta-analysis.

A total of 89 studies included AMR phenotypic susceptibility for 56 different antimicrobial agents (21 antimicrobial classes) among 7 *Enterobacteriaceae* species (Supplementary File 3). *E. coli* and NTS were by far the most common bacterial species (73/89 studies). We selected only studies that reported the AMR prevalence of *E. coli* and NTS in healthy humans, healthy animals/animal food products and environment studies (60/89 studies) for quantitative syntheses (Fig. 3). The detailed description is represented in Supplementary Table 1.

Regarding the highest priority critically important antimicrobials in the three compartments, the highest levels of resistance were observed against 3rd generation of cephalosporins and quinolones. *E. coli*, resistance to ceftriaxone in humans was 56.7% (SE ± 16.5), followed by ceftazidime 30.4% (SE ± 19.7), and ciprofloxacin 27.6% (SE ± 14.4). In animal isolates, resistance levels were higher in quinolones than other drugs in animals, ranging from 28.4–62.5% for *E. coli* and 4.4–22.0% for NTS. For environmental isolates, the prevalence of *E. coli* ranged from 17.8–45.0% to 3rd generation of cephalosporins and 22.7–41.4% to quinolones, while in NTS the resistance prevalence was found highest in quinolones, 7.0–17.9%. Colistin-resistant *E. coli* was found at 34.1% in healthy animals. Neither *E. coli* nor *Salmonella* isolates were resistant to colistin in human studies, and the resistance level to colistin was observed in only NTS in the environment at 4.1% (SE ± 2.8).

With regards to high priority critically important antimicrobials, levels of resistance to ampicillin of *E. coli* and NST were at 67.9–80.2% and 27.9–43.6%; and for gentamicin 18.2–35.0% and 6.4–12.3%, respectively. In carbapenem antimicrobials, the resistance level at 24.4% (SE ± 24.4) was observed in only *E. coli* to meropenem in the environment, and there were no observations of resistance of *Salmonella* to carbapenem class.

For antimicrobials classified as highly important, the resistance prevalence of *E. coli* and NTS to tetracycline in all sectors ranged from 60.9–84.6% and 33.0–60.1%, with the highest level being in animals, followed by humans and the environment. For other drugs, sulfamethoxazole-trimethoprim and chloramphenicol were also found high in all groups, ranging from 54.4–70.8% and 37.4–70.2% in *E. coli*, 23.6–38.9% and 23.1–38.8% in NTS, respectively.

The highest MDR prevalence of *E. coli* was found at 80.4% and 80.0% in animals and humans, respectively. The MDR prevalence of *E. coli* was observed in the environment studies at 52.0%. In contrast, in NTS the highest MDR was observed in the environment (48.9%), followed by animals (47.9%) and humans (23.0%).

### 3.4 Meta-analysis of selected studies

According to the results from the systematic review, only 55 studies representing the resistance levels of *E. coli* and NTS to 6 antimicrobials of ciprofloxacin, ceftazidime, ampicillin, gentamicin, sulfamethoxazole-trimethoprim, chloramphenicol and MDR in the groups of healthy human, healthy animal and environment were used for meta-analysis (Supplementary File 4). The forest plots of pooled prevalence of *E.coli* and NTS to these antimicrobials are shown in Supplementary Fig. 1. A summary of the subgroup meta-analysis of the pooled AMR prevalence across the human, animal and environment studies is represented in Table 3.

In meta-analyses of all studies reported data on 6 selected drugs, the inverse variance indexes (*I*^*2*^) were found high in selected studies of each antimicrobial, ranging from 80.0%–98.0% (all *p* < 0.01), implying that there is significant heterogeneity among studies' results.

**Table 3:** Subgroup meta-analysis of pooled AMR prevalence of *E. coli* and NTS across the human, animal and environment studies.

Antimicrobials	*E. coli*				NTS			
	Human	Animal	Environment	Total	Human	Animal	Environment	Total
Ceftazidime								
Pooled prevalence (%)	25.0	4.0	8.0	6.0⁎	3.0	1.0	2.0	1.0
95% CI	[0−100]	[2.0–6.0]	[0–58.0]	[3.0–15.0]	[0–81.0]	[0–3.0]	[0–16.0]	[0–3.0]
Number of studies (n)	2	7	5	14	2	16	6	24
Ciprofloxacin								
Pooled prevalence (%)	14.0	32.0	24.0	26.0	8.0	6.0	6.0	5.0
95% CI	[0–85.0]	[19.0–49.0]	[12.0–42.0]	[16.0–39.0]	[0–67.0]	[3.0–12.0]	[1.0–31.0]	[3.0–10.0]
Number of studies (n)	4	11	8	23	2	21	6	29
Gentamicin								
Pooled prevalence (%)	17.0	33.0	16.0	25.0	12.0	8.0	8.0	8.0
95% CI	[0–96.0]	[21.0–48.0]	[6.0–34.0]	[16.0 0–37.0]	[1.0–66.0]	[5.0–13.0]	[4.0–16.0]	[5.0–12.0]
Number of studies (n)	3	13	6	22	2	23	9	34
Ampicillin								
Pooled prevalence (%)	88.0	88.0	71.0	84.0	30.0	40.0	17.0	34.0
95% CI	[5.0–100]	[73.0–96.0]	[50.0–86.0]	[73.0–91.0]	[6.0–73.0]	[28.0–54.0]	[3.0–58.0]	[24.0–46.0]
Number of studies (n)	3	13	6	22	2	22	10	34
Chloramphenicol								
Pooled prevalence (%)	39.0	82.0	34.0	65.0⁎⁎	24.0	37.0	18.0	33.0⁎
95% CI	[33.0–46.0]	[51.0–95.0]	[20.0–52.0]	[43.0–82.0]	[4.0–70.0]	[29.0–46.0]	[4.0–56.0]	[25.0–42.0]
Number of studies (n)	3	10	4	17	2	20	9	31
Sulfamethoxazole-trimethoprim								
Pooled prevalence (%)	66.0	73.0	53.0	66.0	25.0	37.0	23.0	33.0
95% CI	[30.0–90.0]	[62.0–82.0]	[34.0–71.0]	[56.0–75.0]	[4.0–71.0]	[27.0–47.0]	[6.0–57.0]	[25.0–42.0]
Number of studies (n)	3	11	8	22	2	18	7	27

⁎ p < 0.05.

⁎⁎ p < 0.01.

The pooled prevalence of *E. coli* and NTS to ciprofloxacin was 26.0% (95% CI 16.0–39.0%) and 5.0% (95% CI 3.0–10.0%), and for ceftazidime they were at 6.0% (95% CI 3.0–15.0%) and 1% (95% CI 0–3.0%). There was a significant difference observed in only ceftazidime-resistant *E. coli*, with the pooled prevalence in humans at 25.0% higher than in animals at 4.0% and environment at 8% (χ^2^ = 8.29, *p* = 0.02). For ampicillin, the pooled prevalence of *E. coli* was high at 84.0% (95% CI 73.0–91.0%) and 34.0% (95% CI 24.0–46.0%) for NTS; and for gentamicin, the pooled prevalence was 25% (95% CI 16.0–37.0%) and 8% (95% CI 5.0–12.0%) in *E. coli* and NTS, respectively. The pooled prevalence of *E. coli* to chloramphenicol and sulfamethoxazole-trimethoprim was high at 65.0% (95% CI 43.0–82.0%) and 66.0% (95% CI 56.0–75.0%) respectively, and for NTS the prevalence was the similar between chloramphenicol and sulfamethoxazole-trimethoprim at 33.0% (95% CI 25.0–42.0%). The significant differences were found with the pooled prevalence in humans, animals and environment of *E. coli* 39.0%, 82.0% and 34.0% (χ^2^ = 9.65, *p* < 0.01) and in NTS 24.0%, 37.0% and 18.0% (χ^2^ = 7.51, *p* = 0.02).

The statistical models investigating the factors associated with the pooled MDR prevalence using meta-regression models are presented in Table 4. In univariable models, all four independent variables (1) bacterial, (2) sectors, (3) study regions and (4) year of publication were selected as potential variables used in multivariable meta-regression models. Findings from the final model of multivariable found that the AMR level in *E. coli* (β = 1.887, *p* < 0.001) and the northern region (β = 0.798, *p* = 0.047) had a higher fraction of AMR than NTS and other regions of Vietnam.

**Table 4:** Multiple meta-regression analyses of studies with data on the pooled prevalence of MDR.

Variables	Univariable models			Multivariable model⁎		
	*β*	95% CI	*p*-value	*β*	95% CI	*p*-value
Bacterial species (baseline = NTS)						
*E. coli*	1.952	1.19–2.71	<0.001	1.887	1.16–2.61	<0.001
Sector (baseline = environment)						
Human	0.139	−1.82-2.10	0.887	0.533	−0.92-1.99	0.462
Animal	0.706	−0.50-1.91	0.147	1.027	1.36–1.92	0.225
Study regions (baseline = Southern region)						
Northern region	1.276	0.24–2.31	0.017	0.798	−0.04-1.64	0.047
Central region	2.140	−0.12-4.40	0.063	0.660	−1.23-2.55	0.485
Multi-region	1.652	−0.20-3.50	0.079	1.370	−0.177-2.916	0.081
Year of publication (baseline = 2006–2010)						
2002–2005	1.77	−1.63-5.18	0.299	−0.253	−2.98-2.48	0.853
2011–2015	0.923	−0.45-2.30	0.184	0.291	−0.76-1.34	0.579
2016–2020	0.628	−0.53-1.79	0.283	0.060	−0.83-0.95	0.892
2021–2022	1.170	−0.27-2.61	0.108	0.876	−0.25-2.00	0.123

⁎ Intercept: -0.904; SE: 0.541.

Analyses for publication bias found the asymmetry among selected articles for meta-analysis. In the contour-enhanced funnel plots, the scatters illustrating selected articles are unevenly distributed far away from the pooled effect size (vertical line) and mostly plotted on the shaded regions of *p* < 0.05 and *p* < 0.01. The findings are confirmed by the results of Egger's regression tests. The selected studies showing the intercept (*β*_*o*_) of selected studies for meta-analyses of ciprofloxacin, ceftazidime and gentamicin, sulfamethoxazole-trimethoprim, chloramphenicol and MDR were different from zero value (all *β*_*o*_ either <−0.72 or > 1.09), whereas in studies reported data on ampicillin, although the intercept of Egger's regression tests was equal to zero, no significance was detected (*p* = 0.998). This indicates that there is the existence of publication bias in the selected studies for meta-analysis. All contour-enhanced funnel plots illustrating the asymmetry among selected studies and the results of Egger's regression tests are shown in Supplementary Fig. 2.

## 4 Discussion

This is the first systematic review and meta-analysis of integrated studies on AMR under the One Health perspective in Vietnam. A total of 89 studies on *Enterobacteriaceae* isolates over three compartments of humans, animals and environment were selected in the review. The sub-groups of healthy and diseased humans and animals were classified. Furthermore, the separation of bacterial species into various subsets and selection of only 55 studies on commensal *E. coli* and NTS in healthy humans and animals for meta-analysis helps to minimize the information bias and accurate appraisal of the associations of antimicrobial phenotypic resistance of *E. coli* and NTS across three compartments.

There were marginal differences in the AMR prevalence of *E. coli* and NTS across the three compartments. The high AMR levels could be stemmed from the transfer of AMR bacteria and AMR-encoding genes due to close contact between humans, animals and the environment [117]. A recent One Health study in Vietnam reported a high similarity in AMR profiles of *E. coli* from humans and chickens from the same farms higher than those from different farms implying the potential transmission of cross-species [118]. Furthermore, the presence of bacterial isolates resistant to antimicrobials commonly administered to humans and animals from wastewater has been demonstrated in Vietnam [71,97]. Of 18 articles regarding environment studies in our review, eight studies included samples collected from wastewater from hospitals and slaughterhouses. This might suggest that AMR bacteria from human and animal sewage are discharged into the environment. There have been demonstrated that bacteria resistant to antimicrobials commonly administered to humans (penicillins, aminoglycosides, quinolones) were present in the hospital wastewater, and seriously there was no difference in AMR levels of isolates that samples collected before and after the treatment of water [69]

The generation of antimicrobial resistance stems from selection pressure caused by the widespread usage of antimicrobials in communities, healthcare settings, and animal production [119]. In terms of antimicrobial use (AMU) of antimicrobial active ingredients per kg of human and animal biomass, the use of antimicrobials in humans and animals in Vietnam was estimated to be 261.7 mg and 247.3 mg in comparison to 122.0 mg and 151.5 mg in the European countries; particularly in the chicken production system the levels of AMU was about 6 times higher with 84.0% usage was for the prophylactic purpose [[17], [19]]. A recent study measured the antimicrobials usage in humans and animals in Vietnam found that the antimicrobials mostly consumed in humans were cephalosporins (49.4%), followed by penicillins (40.0%) and quinolones (9.3%), whereas in animals penicillins (35.5%), tetracyclines (30.7%) and macrolides (19.1%) were commonly given to pigs and chickens [120]. Results about AMR levels in our studies found high AMR levels of *E. coli* and NTS in these drugs showing that the high levels of AMR might be associated with the usage of antimicrobials.

We found similar results as in a review on AMR from Cameroon with levels of *E. coli* resistant to tetracycline (85.5%), trimethoprim-sulfamethoxazole (83.3%), and ampicillin (65.6%) [121]. Another systematic review in Ethiopia conducted in three compartments of humans, animals and environment also observed the highest levels of *E. coli* to ciprofloxacin (50.0%) and ceftriaxone (28.0%) among reviewed drugs [122]. However, this review found a higher prevalence in humans compared with animals and the environment.

In our review, levels of AMR were considerably higher for *E. coli* than for *Salmonella* for almost all antimicrobials. The findings were in agreement with a previous study in chickens and pigs that found that the AMR of *E. coli* was higher than *Salmonella* in all tested antimicrobials [121]. The difference in AMR development among bacterial species is influenced by the ability of selective pressures of a bacterial species under the presence of antimicrobials to be survival than other species. Compared with other bacterial species, *E. coli* has a greater capacity to integrate ARGs from other bacteria and as well as transfer the genes to others [123].

We found that the northern region of Vietnam has higher AMR levels than the other two regions. Most studies in the north were conducted in the Red River Delta, known to have some of the highest densities of human and animal populations in Vietnam [18]. It has been proposed that higher population and animal production densities are correlated with AMU and AMR [124]. Recent studies demonstrated to a high-level detail amounts of antimicrobials used in human and chicken production systems in the south of Vietnam [[7], [125],^118^]. However, there is an existing gap in AMU studies in the northern region. Thus, studies about AMU quantification in different regions in the country are suggested [53].

Following the Global Action Plan on AMR [126], Vietnam also has launched its National Action Plan for the management of AMU and control of AMR, including key activities to support surveillance, improve the awareness, governance and good practices of AMU in humans and animals [127]. Antimicrobials used for growth promotion purposes have been banned in Vietnam since January 2018 [128]. The new legislation on AMU to gradually ban prophylactic use of antimicrobials in animal production systems was also issued in 2020: the critically important antimicrobials were banned from 2021, the highly important from 2022, and the important antimicrobials from 2023 and any other antimicrobial classes from 2026 [129,130]. Since almost selected studies in our reviews were conducted before the time the legislation on the prohibition of prophylactic AMU was released, we suggested that follow-up studies should be conducted in Vietnam to see how well the new regulation policy works in practice.

There were a number of limitations in our study. Given that our review mainly evaluated phenotypic data of AMR in all three compartments of published studies in humans, animals and the environment, the suggestions for the follow-up review on genotypic resistance to have a comprehensive approach to AMR situation in Vietnam. Our review assessed the articles conducted in various geographical locations, mostly the North and the South, and different sample types; the heterogeneity in the study results was therefore found significant in the meta-analysis. Moreover, the high heterogeneity could stem from the publication bias by the effects of small studies in our review. This may result in difficulty in assessing the representativeness of AMR situation in the whole country.

## 5 Conclusion

This review confirms that Vietnam is a hotspot of AMR. Our study revealed the high and similar AMR prevalence of *E. coli* and NTS over the three compartments of humans, animals and the environment. Importantly, bacteria were resistant to several antimicrobials categorized as critically important. Our analyses have yielded some findings on the burden of AMR that should be helpful to baseline information for further investigation to understand the evolution and transmission mechanisms of antimicrobial-resistant bacteria in all three compartments of humans, animals and the environment. Given the observed high prevalence of resistance against the highest critically important antimicrobials, we recommend that their use be restricted, particularly in animal production. Since only a single compartment does not have adequate ability to tackle this global health problem, we strongly recommend implementing AMR surveillance through an integrated collaboration, including multiple scales and levels under the One Health approach to tackle the AMR problem in Vietnam.

The following are the supplementary data related to this article.

Supplementary Fig. 1 Forest plot of pooled prevalence of *E.coli* and NTS to six antimicrobials in meta-analysis. Supplementary Fig. 1

Supplementary Fig. 2 The contour-enhanced funnel plots show the asymmetry among selected studies. The dashed vertical lines show the average effect sizes, each scatter presents each selected article, and the shaded regions illustrate the statistical significance of the asymmetry of the publications. CIP - Ciprofloxacin, CAZ - Ceftazidime, AMP - Ampicillin, GEN- Gentamicin, SXT - Sulfamethoxazole-trimethoprim, CHL-Chloramphenicol, MDR - Multi-drug Resistance. Supplementary Fig. 2

Supplementary File 1 The PRISMA item checklist. Supplementary File 1

Supplementary File 2 A total number of 89 selected studies were used for systematic review and meta-analysis. Supplementary File 2

Supplementary File 3 The data on AMR extracted from 89 selected studies for systematic review Supplementary File 3

Supplementary File 4 The data on AMR extracted from 55 selected studies for meta-analysis Supplementary File 4

Supplementary Table 1 The average AMR prevalence of *E. coli* and Non-typhoidal *Salmonella* (NTS) in healthy humans, healthy animals/ animal food products and the environment. Supplementary Table 1

## Author contributions

DHP, TW, TT, JCM designed the study and developed the protocol. DHP, DBT, TW selected the articles and extracted the data. DHP, DBT contributed to data analyses. DHP, TW, DBT, NVC, TT and JCM contributed to writing up the manuscript. All authors have approved the submitted version of the manuscript.

## Funding

This work has been supported by 10.13039/501100010034Walailak University Graduate Studies Research Fund (No. CGS-RF 2022/09), awarded to Hoang Phu Doan (Doan Hoang Phu).

## Conflicts of interest

All authors declare no conflict of interest.

## Data Availability

The research data are available in supplementary materials.
