# Supplementary material for: A systematic review and meta-analysis of integrated studies on antimicrobial resistance in Vietnam, with a focus on Enterobacteriaceae, from a One Health perspective
Source: One Health. 2022 Nov 19;15:100465. doi: 10.1016/j.onehlt.2022.100465 (PMC9767812; doi:10.1016/j.onehlt.2022.100465)

Supplementary Figure 1: Forest plot of the pooled prevalence of *E. coli* (left side, in orange) and NTS (right side, in yellow) to six antimicrobials in meta-analysis

1. Ceftadizime

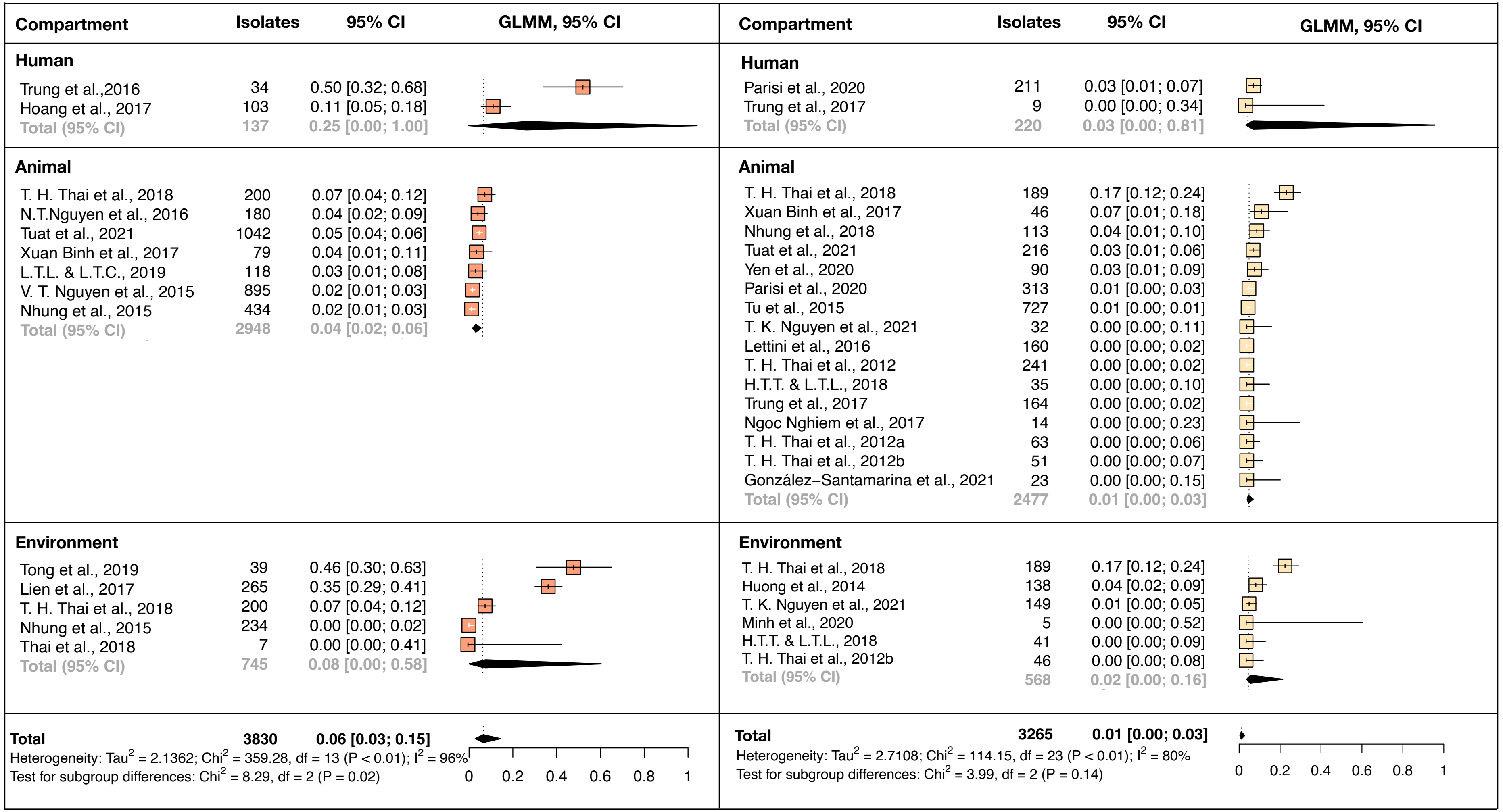

2. Ciprofloxacin

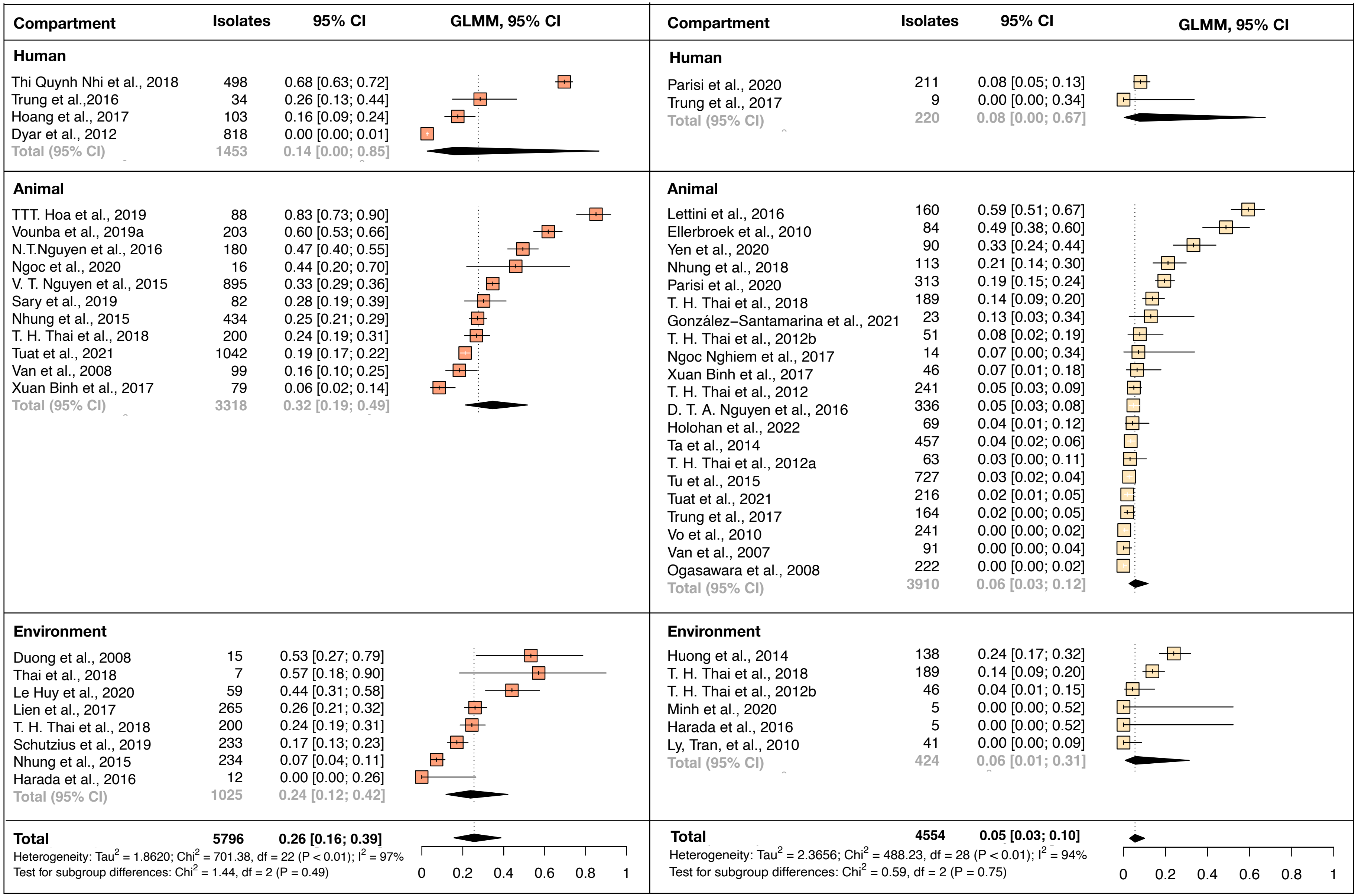

3. Gentamicin

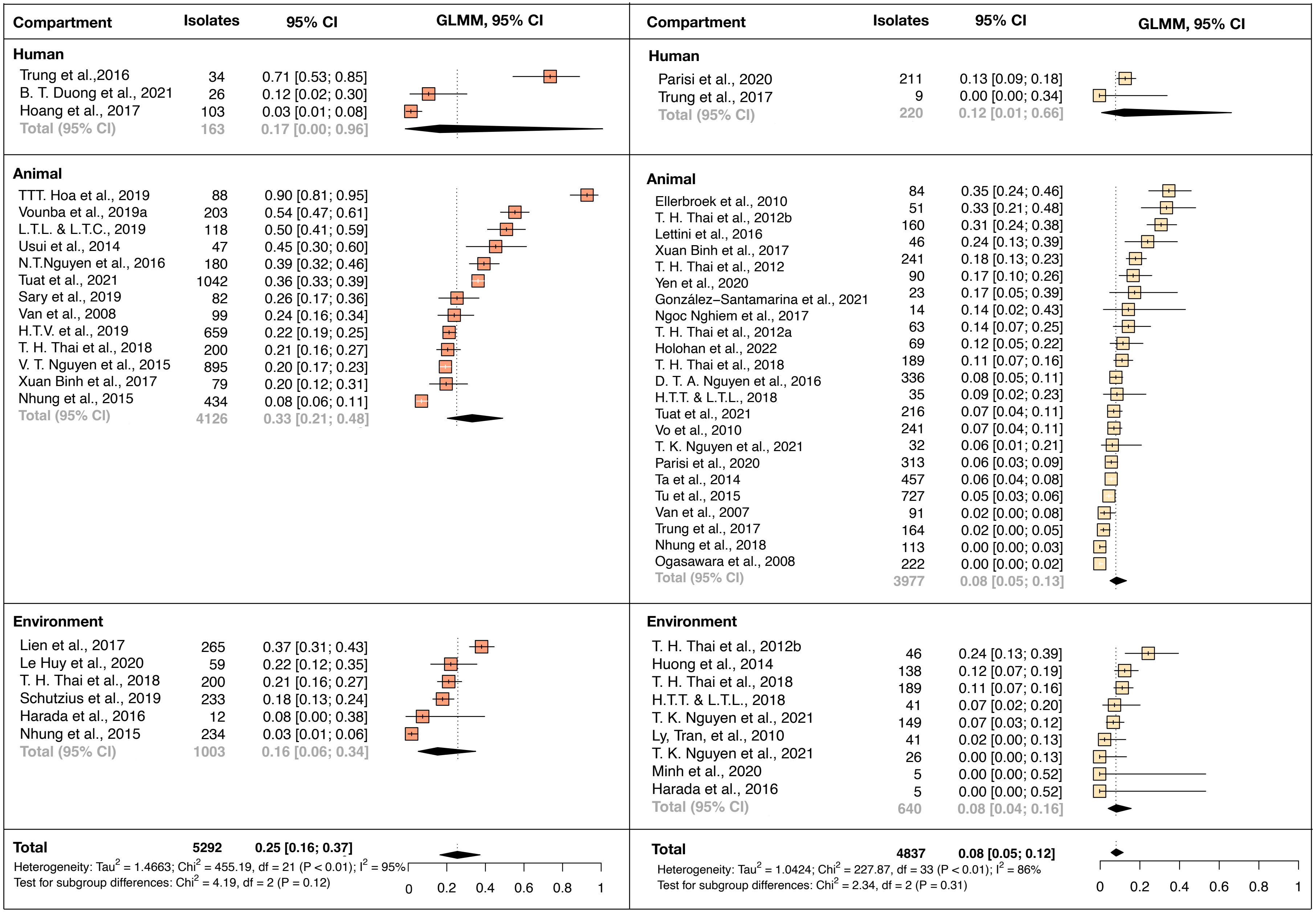

4. Ampicillin

Compartment	Isolates	95% CI
**Human**		
Trung et al.,2016	34	1.00 [0.90; 1.00]
Hoang et al., 2017	103	0.70 [0.60; 0.79]
Dyar et al., 2012	818	0.70 [0.67; 0.73]
Total (95% CI)	955	0.88 [0.05; 1.00]
**Animal**		
Vounba et al., 2019a	203	1.00 [0.98; 1.00]
N.T.Nguyen et al., 2016	180	1.00 [0.98; 1.00]
V. T. Nguyen et al., 2015	895	0.90 [0.88; 0.92]
Tuat et al., 2021	1042	0.90 [0.88; 0.92]
L.T.L. & L.T.C., 2019	118	0.90 [0.83; 0.95]
Kha et al., 2006	87	0.90 [0.81; 0.95]
Nguyen Tat Toan, 2021	90	0.90 [0.82; 0.95]
Nhung et al., 2015	434	0.80 [0.76; 0.84]
Usui et al., 2014	47	0.81 [0.67; 0.91]
Sary et al., 2019	82	0.70 [0.58; 0.79]
H.T.V. et al., 2019	659	0.60 [0.56; 0.64]
T. H. Thai et al., 2018	200	0.60 [0.53; 0.67]
Van et al., 2008	99	0.51 [0.40; 0.61]
Total (95% CI)	4136	0.88 [0.73; 0.96]
**Environment**		
Nhung et al., 2015	234	0.90 [0.86; 0.94]
Le Huy et al., 2020	59	0.80 [0.67; 0.89]
Tong et al., 2019	39	0.79 [0.64; 0.91]
Schutzius et al., 2019	233	0.60 [0.53; 0.66]
T. H. Thai et al., 2018	200	0.60 [0.53; 0.67]
Harada et al., 2016	12	0.33 [0.10; 0.65]
Total (95% CI)	777	0.71 [0.50; 0.86]
**Total**	**5868**	**0.84 [0.73; 0.91]**

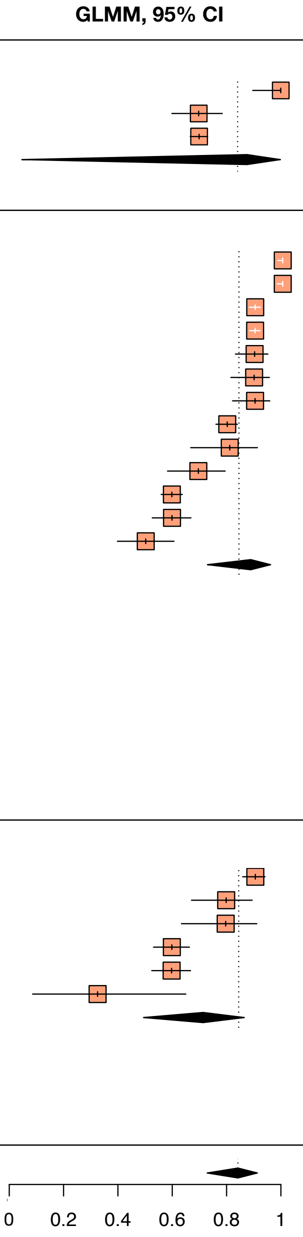

Heterogeneity: $Tau^2 = 2.1768$; $Chi^2 = 475.19$, df = 21 ($P < 0.01$); $I^2 = 96\%$
Test for subgroup differences: $Chi^2 = 3.80$, df = 2 ($P = 0.15$)

Compartment	Isolates	95% CI
**Human**		
Parisi et al., 2020	211	0.30 [0.24; 0.37]
Trung et al., 2017	9	0.22 [0.03; 0.60]
Total (95% CI)	220	0.30 [0.06; 0.73]
**Animal**		
Tuat et al., 2021	216	0.90 [0.85; 0.94]
González–Santamarina et al., 2021	23	0.70 [0.47; 0.87]
Yen et al., 2020	90	0.60 [0.49; 0.70]
T. H. Thai et al., 2018	189	0.60 [0.52; 0.67]
Holohan et al., 2022	69	0.59 [0.47; 0.71]
Lettini et al., 2016	160	0.50 [0.42; 0.58]
Nhung et al., 2018	113	0.50 [0.41; 0.60]
H.T.T. & L.T.L., 2018	35	0.51 [0.34; 0.69]
Ngoc Nghiem et al., 2017	14	0.50 [0.23; 0.77]
T. H. Thai et al., 2012b	51	0.51 [0.37; 0.65]
T. K. Nguyen et al., 2021	32	0.50 [0.32; 0.68]
Ta et al., 2014	457	0.40 [0.36; 0.45]
D. T. A. Nguyen et al., 2016	336	0.40 [0.35; 0.45]
T. H. Thai et al., 2012	241	0.40 [0.34; 0.46]
Ellerbroek et al., 2010	84	0.40 [0.30; 0.52]
Vo et al., 2010	241	0.30 [0.24; 0.36]
Trung et al., 2017	164	0.30 [0.23; 0.38]
T. H. Thai et al., 2012a	63	0.30 [0.19; 0.43]
Parisi et al., 2020	313	0.20 [0.16; 0.25]
Tu et al., 2015	727	0.20 [0.17; 0.23]
Van et al., 2007	91	0.20 [0.12; 0.29]
Ogasawara et al., 2008	222	0.00 [0.00; 0.02]
Total (95% CI)	3931	0.40 [0.28; 0.54]
**Environment**		
Huong et al., 2014	138	0.60 [0.51; 0.68]
T. H. Thai et al., 2018	189	0.60 [0.52; 0.67]
H.T.T. & L.T.L., 2018	41	0.51 [0.35; 0.67]
T. H. Thai et al., 2012b	46	0.50 [0.35; 0.65]
T. K. Nguyen et al., 2021	149	0.50 [0.42; 0.59]
D. T. A. Nguyen et al., 2021	102	0.30 [0.22; 0.40]
T. K. Nguyen et al., 2021	26	0.00 [0.00; 0.13]
Minh et al., 2020	5	0.00 [0.00; 0.52]
Harada et al., 2016	5	0.00 [0.00; 0.52]
Ly, Tran, et al., 2010	41	0.00 [0.00; 0.09]
Total (95% CI)	742	0.17 [0.03; 0.58]
**Total**	**4893**	**0.34 [0.24; 0.46]**

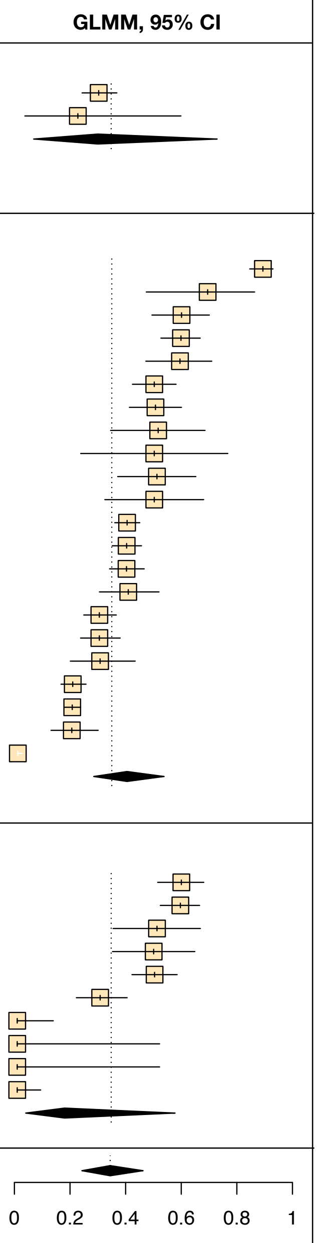

Heterogeneity: $Tau^2 = 1.7757$; $Chi^2 = 480.47$, df = 33 ($P < 0.01$); $I^2 = 93\%$
Test for subgroup differences: $Chi^2 = 3.26$, df = 2 ($P = 0.20$)

5. Chloramphenicol

Compartment	Isolates	95% CI
**Human**		
Dyar et al., 2012	818	0.40 [0.37; 0.43]
Trung et al.,2016	34	0.38 [0.22; 0.56]
Hoang et al., 2017	103	0.34 [0.25; 0.44]
Total (95% CI)	955	0.39 [0.33; 0.46]
**Animal**		
Kha et al., 2006	87	1.00 [0.96; 1.00]
Nguyen Tat Toan, 2021	90	1.00 [0.96; 1.00]
Vounba et al., 2019a	203	0.86 [0.81; 0.91]
Tuat et al., 2021	1042	0.86 [0.84; 0.88]
TTT. Hoa et al., 2019	88	0.78 [0.68; 0.86]
V. T. Nguyen et al., 2015	895	0.68 [0.65; 0.71]
Usui et al., 2014	47	0.51 [0.36; 0.66]
Sary et al., 2019	82	0.49 [0.38; 0.60]
Van et al., 2008	99	0.43 [0.33; 0.54]
Nhung et al., 2015	434	0.40 [0.35; 0.45]
Total (95% CI)	3067	0.82 [0.51; 0.95]
**Environment**		
Le Huy et al., 2020	59	0.46 [0.33; 0.59]
Harada et al., 2016	12	0.42 [0.15; 0.72]
Schutzius et al., 2019	233	0.37 [0.31; 0.43]
Nhung et al., 2015	234	0.23 [0.17; 0.29]
Total (95% CI)	538	0.34 [0.20; 0.52]
**Total (95% CI)**	**4560**	**0.65 [0.43; 0.82]**

GLMM, 95% CI

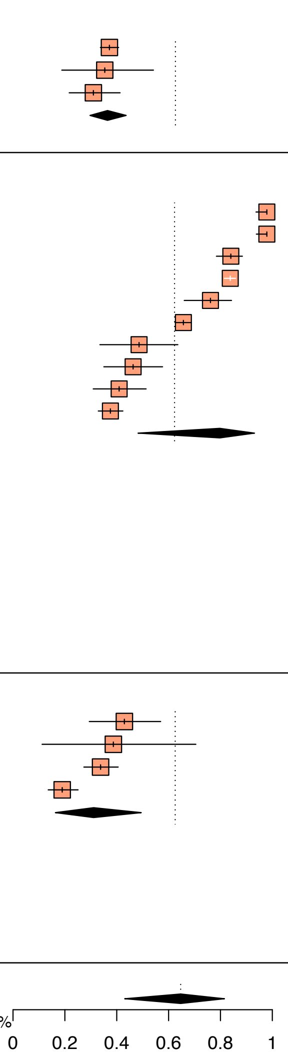

Heterogeneity: Tau^2 = 2.7683; Chi^2 = 721.40, df = 16 (P < 0.01); I^2 = 98%
Test for subgroup differences: Chi^2 = 9.65, df = 2 (P < 0.01)

Compartment	Isolates	95% CI
**Human**		
Parisi et al., 2020	211	0.24 [0.19; 0.31]
Trung et al., 2017	9	0.22 [0.03; 0.60]
Total (95% CI)	220	0.24 [0.04; 0.70]
**Animal**		
Tuat et al., 2021	216	0.76 [0.70; 0.81]
T. K. Nguyen et al., 2021	32	0.62 [0.44; 0.79]
Ngoc Nghiem et al., 2017	14	0.57 [0.29; 0.82]
Holohan et al., 2022	69	0.57 [0.44; 0.68]
Lettini et al., 2016	160	0.53 [0.45; 0.61]
H.T.T. & L.T.L., 2018	35	0.51 [0.34; 0.69]
Nhung et al., 2018	113	0.48 [0.38; 0.57]
Yen et al., 2020	90	0.42 [0.32; 0.53]
Ellerbroek et al., 2010	84	0.42 [0.31; 0.53]
T. H. Thai et al., 2012b	51	0.41 [0.28; 0.56]
D. T. A. Nguyen et al., 2016	336	0.38 [0.32; 0.43]
T. H. Thai et al., 2012	241	0.37 [0.31; 0.44]
Ta et al., 2014	457	0.35 [0.30; 0.39]
Trung et al., 2017	164	0.28 [0.21; 0.36]
Tu et al., 2015	727	0.27 [0.24; 0.31]
Parisi et al., 2020	313	0.23 [0.18; 0.28]
T. H. Thai et al., 2012a	63	0.22 [0.13; 0.34]
Vo et al., 2010	241	0.19 [0.14; 0.24]
Ogasawara et al., 2008	222	0.12 [0.08; 0.17]
Noor Uddin et al., 2015	18	0.06 [0.00; 0.27]
Total (95% CI)	3646	0.37 [0.29; 0.46]
**Environment**		
T. K. Nguyen et al., 2021	149	0.63 [0.55; 0.71]
Huong et al., 2014	138	0.54 [0.45; 0.62]
H.T.T. & L.T.L., 2018	41	0.54 [0.37; 0.69]
T. H. Thai et al., 2012b	46	0.43 [0.29; 0.59]
D. T. A. Nguyen et al., 2021	102	0.34 [0.25; 0.44]
Ly, Tran, et al., 2010	41	0.02 [0.00; 0.13]
T. K. Nguyen et al., 2021	26	0.00 [0.00; 0.13]
Minh et al., 2020	5	0.00 [0.00; 0.52]
Harada et al., 2016	5	0.00 [0.00; 0.52]
Total (95% CI)	553	0.18 [0.04; 0.56]
**Total (95% CI)**	**4419**	**0.33 [0.25; 0.42]**

GLMM, 95% CI

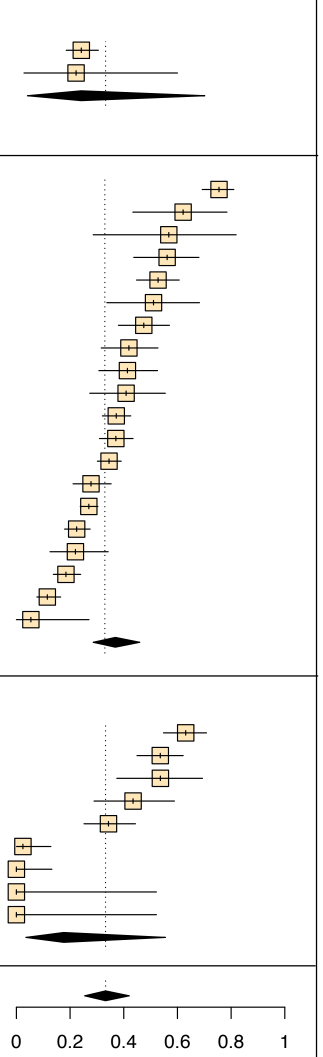

Heterogeneity: Tau^2 = 0.8893; Chi^2 = 402.70, df = 30 (P < 0.01); I^2 = 93%
Test for subgroup differences: Chi^2 = 7.51, df = 2 (P = 0.02)

6. Sulfamethoxazole-trimethoprim

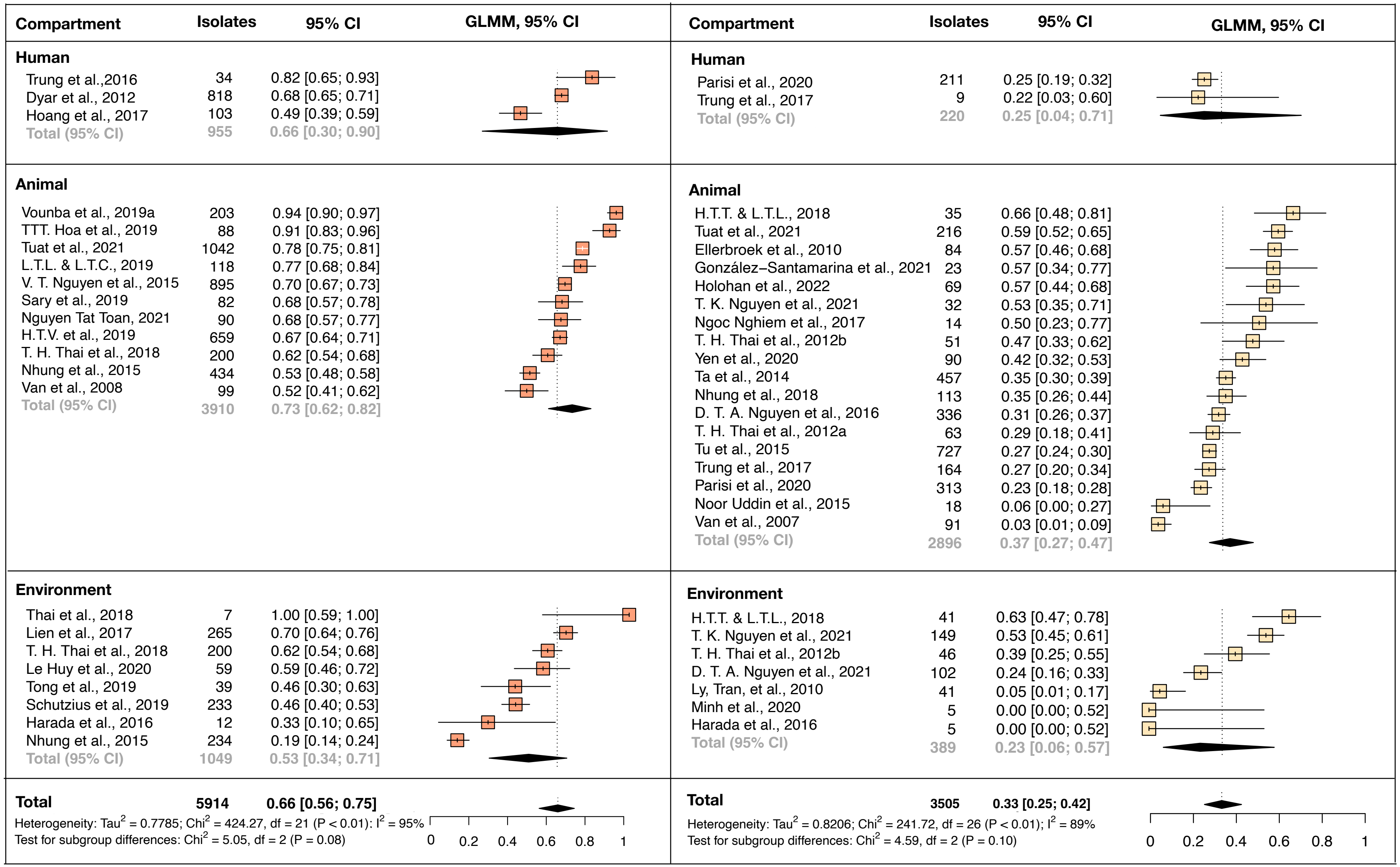

Supplement: Supplementary Fig. 1 — Forest plot of pooled prevalence of E.coli and NTS to six antimicrobials in meta-analysis. [file mmc1.pdf]
