# Supplementary material for: A systematic review and meta-analysis of integrated studies on antimicrobial resistance in Vietnam, with a focus on Enterobacteriaceae, from a One Health perspective
Source: One Health. 2022 Nov 19;15:100465. doi: 10.1016/j.onehlt.2022.100465 (PMC9767812; doi:10.1016/j.onehlt.2022.100465)
Supplement: Supplementary Fig. 2 — The contour-enhanced funnel plots show the asymmetry among selected studies. The dashed vertical lines show the average effect sizes, each scatter presents each selected article, and the shaded regions illustrate the statistical significance of the asymmetry of the publications. CIP - Ciprofloxacin, CAZ - Ceftazidime, AMP - Ampicillin, GEN- Gentamicin, SXT - Sulfamethoxazole-trimethoprim, CHL-Chloramphenicol, MDR - Multi-drug Resistance. [file mmc2.pdf]

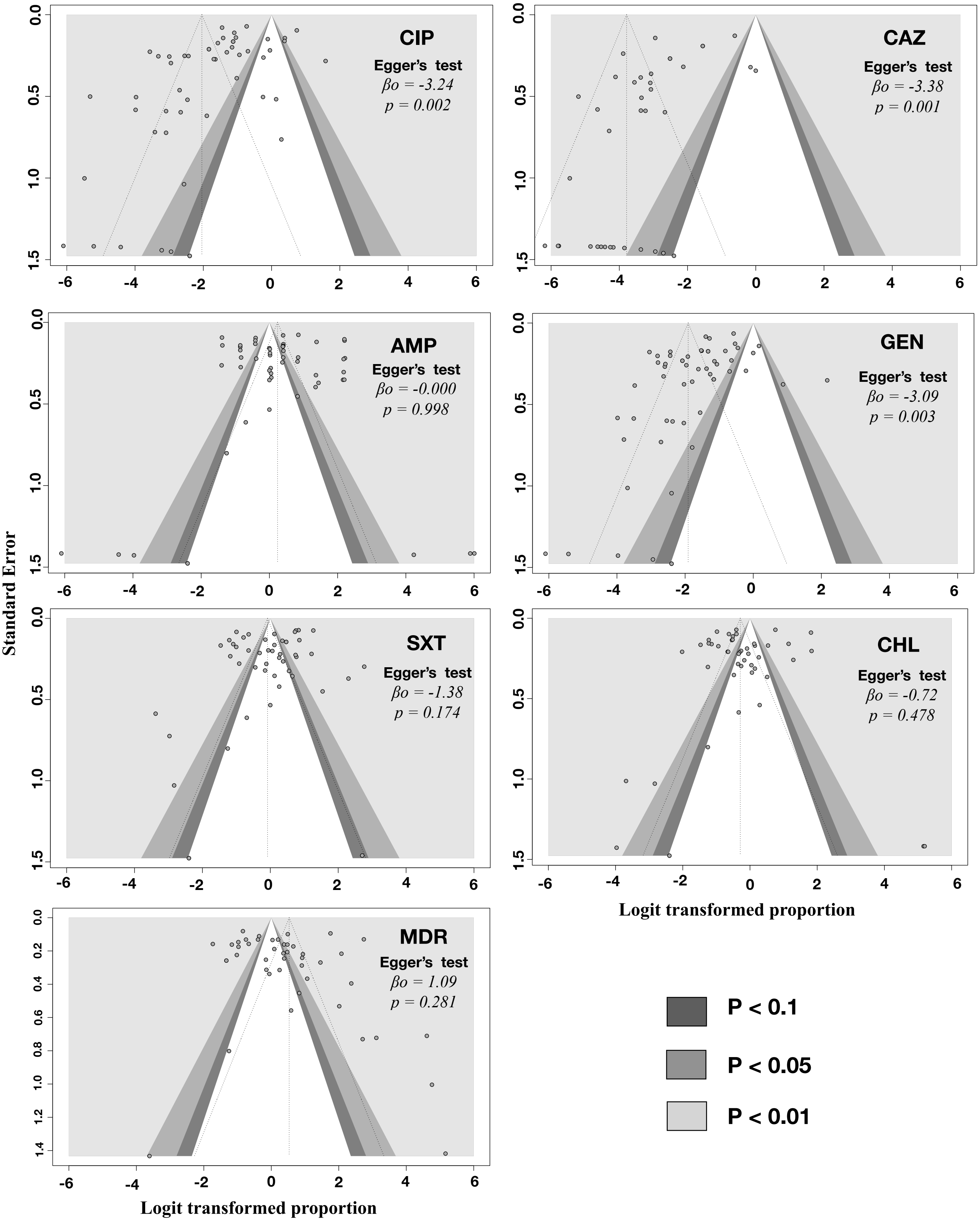

Supplementary Figure 2. The contour-enhanced funnel plots show the asymmetry among selected studies. The dashed vertical lines show the average effect sizes, each scatter presents each selected paper, and the shaded regions illustrate the statistical significance of asymmetry of the publications. CIP - Ciprofloxacin, CAZ - Ceftazidime, AMP - ampicillin, GEN- Gentamicin, SXT - Sulfamethoxazole-trimethoprim, CHL- Chloramphenicol, MDR - Multi-drug Resistance.
