# Supplementary material for: A systematic review and meta-analysis of integrated studies on antimicrobial resistance in Vietnam, with a focus on Enterobacteriaceae, from a One Health perspective
Source: One Health. 2022 Nov 19;15:100465. doi: 10.1016/j.onehlt.2022.100465 (PMC9767812; doi:10.1016/j.onehlt.2022.100465)
Supplement: Supplementary Table 1 — The average AMR prevalence of E. coli and Non-typhoidal Salmonella (NTS) in healthy humans, healthy animals/ animal food products and the environment. [file mmc7.docx]

**Supplementary Table 1.** The average AMR prevalence of *E. coli* and Non-typhoidal *Salmonella* (NTS) in healthy human, healthy animal/ animal food products and the environment.

| **Antimicrobial class** | Bacteria |  | Compartments (% ± SE) (n) | | | | |  |
| --- | --- | --- | --- | --- | --- | --- | --- | --- |
| Antimicrobial agent |  |  | Healthy |  | Healthy animals/  animal products |  | Environment |  |
| **Cephalosporins (3^rd^, 4^th^ and 5^th^ generation)^1^** |  |  |  |  |  |  |  |  |
| Cefepime | *E. coli* |  | 18.0 ± nc (1) |  | _ |  | _ |  |
|  | NTS |  | _ |  | _ |  | _ |  |
| Cefixime | *E. coli* |  | _ |  | _ |  | _ |  |
|  | NTS |  | _ |  | 4.2 ± 2.1 (3) |  | _ | |
| Cefodizime | *E. coli* |  | _ |  | _ |  | 23.7 ± nc (1) |  |
|  | NTS |  | _ |  | _ |  | _ |  |
| Cefoperazone | *E. coli* |  | _ |  | _ |  | _ |  |
|  | NTS |  | _ |  | _ |  | _ |  |
| Cefotaxime | *E. coli* |  | 15.5 ± nc (1) |  | 16.8 ± 4.3 (2) |  | 24.9 ± 16.2 (4) |  |
|  | NTS |  | _ |  | 6.0 ± 2.9 (7) |  | 1.4 ± 1.4 (3) |  |
| Cefpodoxime | *E. coli* |  | _ |  | 4.3 ± nc (1) |  | _ |  |
|  | NTS |  | _ |  | _ |  | _ |  |
| Ceftazidime | *E. coli* |  | 30.4 ± 19.7 (2*) |  | 4.0 ± 0.8 (7*) |  | 17.8 ± 9.6 (5*) |  |
|  | NTS |  | 1.7 ± 1.7 (2*) |  | 2.3 ± 1.1 (16*) |  | 3.8 ± 2.8 (6*) |  |
| Ceftiofur | *E. coli* |  | _ |  | 11.7 ± 5.4 (5) |  | _ |  |
|  | NTS |  | _ |  | 21.0 ± nc (1) |  | _ |  |
| Ceftriaxone | *E. coli* |  | 56.7 ± 16.5 (3) |  | 3.5 ± 0.5 (3) |  | 45.0 ± nc (1) |  |
|  | NTS |  | 1.4 ± 1.4 (2) |  | 2.3 ± 1.0 (8) |  | 0.0 ± nc (1) |  |
| **Glycopeptides^1^** |  |  |  |  |  |  |  |  |
| Vancomycin | *E. coli* |  | _ |  | 100.0 ± nc (1) |  | _ |  |
|  | NTS |  | _ |  | 100.0 ± nc (1) |  | _ |  |
| **Macrolides^1^** |  |  |  |  |  |  |  |  |
| Azithromycin | *E. coli* |  | _ |  | 15.0 ± 6.0 (2) |  | _ |  |
|  | NTS |  | _ |  | 16.0 ± nc (1) |  | 0.0 ± nc (1) |  |
| Erythromycin | *E. coli* |  | _ |  | 15.2 ± nc (1) |  | _ |  |
|  | NTS |  | _ |  | 19.6 ± nc (1) |  | _ |  |
| **Polymyxins^1^** |  |  |  |  |  |  |  |  |
| Colistin | *E. coli* |  | _ |  | 34.1 ± 12.3 (9) |  | _ |  |
|  | NTS |  | _ |  | 5.4 ± 2.2 (10) |  | 4.1 ± 2.8 (4) |  |
| **Quinolones^1^** |  |  |  |  |  |  |  |  |
| Ciprofloxacin* | *E. coli* |  | 27.6 ± 14.4 (4*) |  | 35.0 ± 6.6 (11*) |  | 27.7 ± 7.0 (8*) |  |
|  | NTS |  | 4.1 ± 4.1 (2*) |  | 12.3 ± 3.6 (21*) |  | 7.0 ± 4.0 (6*) |  |
| Enrofloxacin | *E. coli* |  | _ |  | 40.8 ± 10.9 (4) |  | _ |  |
|  | NTS |  | _ |  | 6.7 ± 2.2 (2) |  | _ |  |
| Flumequine | *E. coli* |  | _ |  | 62.5 ± nc (1) |  | _ |  |
|  | NTS |  | _ |  | _ |  | _ |  |
| Levofloxacin | *E. coli* |  | _ |  | 47.5 ± nc (1) |  | _ |  |
|  | NTS |  | _ |  | 11.3 ± 4.4 (4) |  | 4.7 ± 4.7 (2) |  |
| Nalidixic acid | *E. coli* |  | 35.4 ± 8.4 (2) |  | 51.5 ± 10.3 (9) |  | 41.4 ± 21.0 (3) |  |
|  | NTS |  | _ |  | 22.0 ± 2.8 (16) |  | 17.9 ± 6.5 (8) |  |
| Norfloxacin | *E. coli* |  | _ |  | 28.4 ± 5.6 (4) |  | _ |  |
|  | NTS |  | _ |  | 4.4 ± 3.9 (4) |  | 15.9 ± nc (1) |  |
| Ofloxacin | *E. coli* |  | 22.0 ± nc (1) |  | _ |  | _ |  |
|  | NTS |  | _ |  | 20.6 ± 10.6 (5) |  | 3.4 ± 0.6 (2) |  |
| **Aminoglycosides^2^** |  |  |  |  |  |  |  |  |
| Amikacin | *E. coli* |  | 2.9 ± nc (1) |  | 2.3 ± 1.2 (4) |  | _ |  |
|  | NTS |  | 5.0 ± 5.0 (2) |  | 1.6 ± 0.9 (7) |  | 1.6 ± 1.0 (2) |  |
| Apramycin | *E. coli* |  | _ |  | _ |  | _ |  |
|  | NTS |  | _ |  | _ |  | _ |  |
| Dihydrostreptomycin | *E. coli* |  | _ |  | 82.9 ± nc (1) |  | _ |  |
|  | NTS |  | _ |  | _ |  | _ |  |
| Gentamicin* | *E. coli* |  | 28.2 ± 21.4 (3*) |  | 35.0 ± 5.9 (13*) |  | 18.2 ± 4.8 (6*) |  |
|  | NTS |  | 6.4 ± 6.4 (2*) |  | 12.3 ± 2.1 (23*) |  | 7.1 ± 2.6 (9*) |  |
| Kanamycin | *E. coli* |  | 12.6 ± nc (1) |  | 37.1 ± 11.3 (6) |  | 0.0 ± nc (1) |  |
|  | NTS |  | _ |  | 15.3 ± 3.9 (11) |  | 19.6 ± 9.5 (5) |  |
| Neomycin | *E. coli* |  | _ |  | _ |  | _ |  |
|  | NTS |  | _ |  | 19.7 ± 11.8 (2) |  | 28.3 ± nc (1) |  |
| Streptomycin | *E. coli* |  | 80.6 ± nc (1) |  | 69.2 ± 4.8 (9) |  | 47.8 ± 15.1 (3) |  |
|  | NTS |  | _ |  | 36.9 ± 6.4 (13) |  | 37.0 ± 12.0 (8) |  |
| Tobramycin | *E. coli* |  | _ |  | _ |  | _ |  |
|  | NTS |  | _ |  | 9.2 ± 8.3 (2) |  | _ |  |
| **Ansamycins^2^** |  |  |  |  |  |  |  |  |
| Rifampicin | *E. coli* |  | _ |  | 6.3 ± nc (1) |  | _ |  |
|  | NTS |  | _ |  | 8.7 ± nc (1) |  | _ |  |
| **Carbapenems and other penems^2^** |  |  |  |  |  |  |  |  |
| Doripenem | *E. coli* |  | _ |  | _ |  | _ |  |
|  | NTS |  | _ |  | _ |  | _ |  |
| Ertapenem | *E. coli* |  | _ |  | _ |  | 0.0 ± nc (1) |  |
|  | NTS |  | _ |  | 0.0 ± 0.0 (3) |  | _ |  |
| Imipenem | *E. coli* |  | 0.0 ± nc (1) |  | 4.4 ± nc (1) |  | 1.0 ± 0.6 (3) |  |
|  | NTS |  | _ |  | 0.0 ± 0.0 (3) |  | _ |  |
| Meropenem | *E. coli* |  | 0.0 ± nc (1) |  | 3.1 ± 2.0 (3) |  | 24.4 ± 24.4 (2) |  |
|  | NTS |  | 0.0 ± nc (1) |  | 0.0 ± 0.0 (5) |  | 0.0 ± nc (1) |  |
| **Penicillins (antipseudomonal)^2^** |  |  |  |  |  |  |  |  |
| Piperacillin-tazobactam | *E. coli* |  | 2.0 ± nc (1) |  | 60.0 ± nc (1) |  | _ |  |
|  | NTS |  | _ |  | 69.6 ± nc (1) |  | _ |  |
| Ticarcillin + clavulanic acid | *E. coli* |  | 30.0 ± nc (1) |  | _ |  | _ |  |
|  | NTS |  | _ |  | _ |  | _ |  |
| **Penicillins (aminopenicillins)^2^** |  |  |  |  |  |  |  |  |
| Amoxicillin | *E. coli* |  | 50.0 ± nc (1) |  | 78.7 ± 14.7 (3) |  | _ |  |
|  | NTS |  | _ |  | 61.0 ± 39.0 (2) |  | _ |  |
| Ampicillin* | *E. coli* |  | 76.7 ± 11.7 (3*) |  | 80.2 ± 3.9 (13*) |  | 67.9 ± 7.8 (6*) |  |
|  | NTS |  | 27.9 ± 5.9 (2*) |  | 43.6 ± 4.0 (22*) |  | 29.5 ± 8.0 (10*) |  |
| **Penicillins (aminopenicillins with beta- lactamase inhibitors)^2^** |  |  |  |  |  |  |  |  |
| Amoxicillin + clavulanic acid | *E. coli* |  | 50.0 ± nc (1) |  | 18.9 ± 6.4 (8) |  | 29.8 ± 3.9 (3) |  |
|  | NTS |  | 10.0 ± nc (1) |  | 6.2 ± 2.2 (12) |  | 10.2 ± 7.4 (3) |  |
| Ampicillin-sulbactam | *E. coli* |  | _ |  | _ |  | _ |  |
|  | NTS |  | _ |  | _ |  | _ |  |
| **Phosphonic acid derivatives^2^** |  |  |  |  |  |  |  |  |
| Fosfomycin | *E. coli* |  | 4.9 ± nc (1) |  | 6.4 ± nc (1) |  | 3.9 ± 2.2 (3) |  |
|  | NTS |  | _ |  | 0.8 ± 0.8 (2) |  | 0.0 ± nc (1) |  |
| **Amphenicols^3^** |  |  |  |  |  |  |  |  |
| Chloramphenicol* | *E. coli* |  | 37.4 ± 1.8 (3*) |  | 70.2 ± 7.3 (10*) |  | 37.8 ± 5.1 (4*) |  |
|  | NTS |  | 23.1 ± 1.1 (2*) |  | 38.8 ± 4.0 (20*) |  | 27.7 ± 9.0 (9*) |  |
| Florfenicol | *E. coli* |  | _ |  | 20.3 ± nc (1) |  | _ |  |
|  | NTS |  | _ |  | 18.5 ± 10.3 (4) |  | 34.5 ± 19.1 (2) |  |
| **Cephalosporins (1^st^, 2^nd^ generation)^3^** |  |  |  |  |  |  |  |  |
| Cefalotin | *E. coli* |  | _ |  | 9.2 ± 4.9 (2) |  | 32.0 ± nc (1) |  |
|  | NTS |  | _ |  | _ |  | _ |  |
| Cefazolin | *E. coli* |  | _ |  | _ |  | _ |  |
|  | NTS |  | _ |  | 0.0 ± nc (1) |  | 0.0 ± nc (1) |  |
| Cefoxitin | *E. coli* |  | _ |  | 3.0 ± nc (1) |  | 0.0 ± nc (1) |  |
|  | NTS |  |  |  | 2.0 ± 1.3 (3) |  | 0.0 ± nc (1) |  |
| Cefuroxime | *E. coli* |  | _ |  | 9.0 ± 6.5 (2) |  | _ |  |
|  | NTS |  | _ |  | 12.2 ± 6.0 (6) |  | 21.2 ± 18.6 (2) |  |
| **Folate pathway inhibitors^3^** |  |  |  |  |  |  |  |  |
| Sulfamethoxazone -trimethoprime* | *E. coli* |  | 66.7 ± 10.1 (3*) |  | 70.8 ± 4.1 (11*) |  | 54.4 ± 8.7 (8*) |  |
|  | NTS |  | 23.6 ± 1.6 (2*) |  | 38.9 ± 4.3 (18*) |  | 26.4 ± 10.0 (7*) |  |
| **Penicillins (anti- staphylococcal)^3^** |  |  |  |  |  |  |  |  |
| Oxacillin | *E. coli* |  | _ |  | 100.0 ± nc (1) |  | _ |  |
|  | NTS |  | _ |  | 100 ± nc (1) |  | _ |  |
| **Sulfonamides^3^** |  |  |  |  |  |  |  |  |
| Sulfafurazole | *E. coli* |  | _ |  | 78.1 ± 9.8 (3) |  | _ |  |
|  | NTS |  | _ |  | 16.5 ± nc (1) |  | _ |  |
| **Tetracyclines^3^** |  |  |  |  |  |  |  |  |
| Doxycycline | *E. coli* |  | _ |  | 20.1 ± 4.5 (2) |  | _ |  |
|  | NTS |  | _ |  | 9.4 ± 2.8 (2) |  | 9.4 ± 2.8 (2) |  |
| Oxytetracycline | *E. coli* |  | _ |  | 65.9 ± 32.3 (3) |  | _ |  |
|  | NTS |  | _ |  | 8.4 ± 8.4 (2) |  | 29.3 ± 29.3 (2) |  |
| Tetracycline | *E. coli* |  | 70.5 ± 2.0 (3) |  | 84.6 ± 5.9 (10) |  | 60.9 ± 8.0 (6) |  |
|  | NTS |  | 33.0 ± nc (1) |  | 60.1 ± 3.9 (19) |  | 38.5 ± 10.8 (8) |  |
| **Aminocyclitols^4^** |  |  |  |  |  |  |  |  |
| Spectinomycin | *E. coli* |  | _ |  | _ |  | _ |  |
|  | NTS |  | _ |  | _ |  | _ |  |
| **Nitrofurans derivatives^4^** |  |  |  |  |  |  |  |  |
| Nitrofurantoin | *E. coli* |  | _ |  | 2.5 ± nc (1) |  | _ |  |
|  | NTS |  | _ |  | 2.2 ± nc (1) |  | _ |  |
| **Polypeptides^4^** |  |  |  |  |  |  |  |  |
| Bacitracin | *E. coli* |  | _ |  | 48.1 ± nc (1) |  | _ |  |
|  | NTS |  | _ |  | 63.0 ± nc (1) |  | _ |  |
| **MDR*** | *E. coli* |  | 80.0 ± 8.2 (2*) |  | 80.4 ± 6.1 (12*) |  | 52.0 ± 9.7 (5*) |  |
|  | NTS |  | 23.0 ± 0.8 (2*) |  | 47.9 ± 5.4 (20*) |  | 48.9 ± 8.4 (4*) |  |

^1^highest priority critically important antimicrobials, ^2^high priority critically important antimicrobials, ^3^highly important antimicrobials, ^4^important antimicrobials, * antimicrobials of studies used for meta-analysis.
